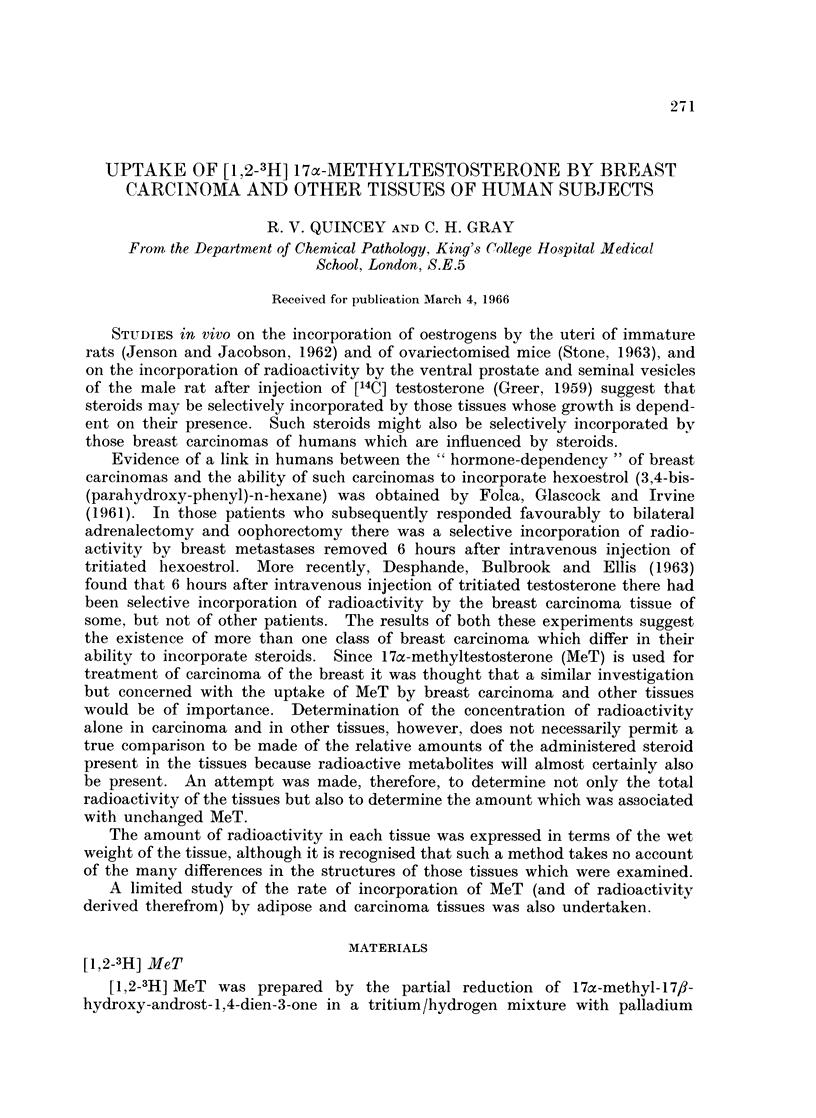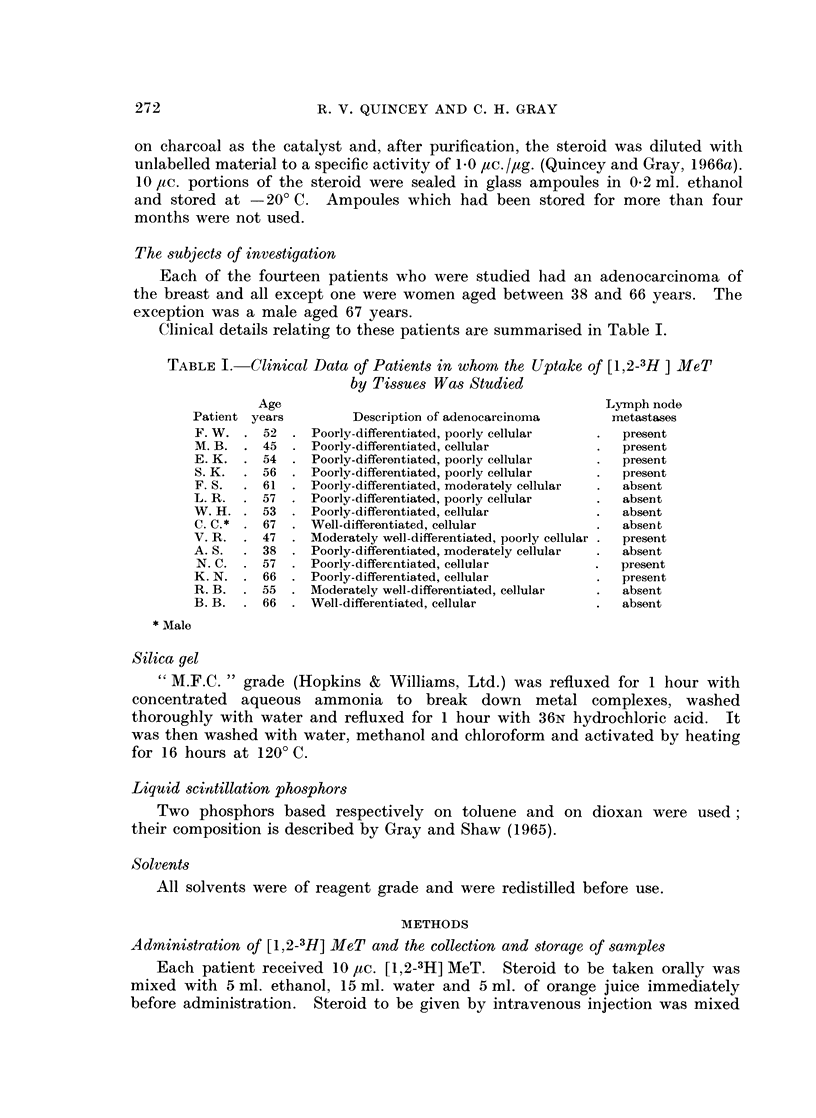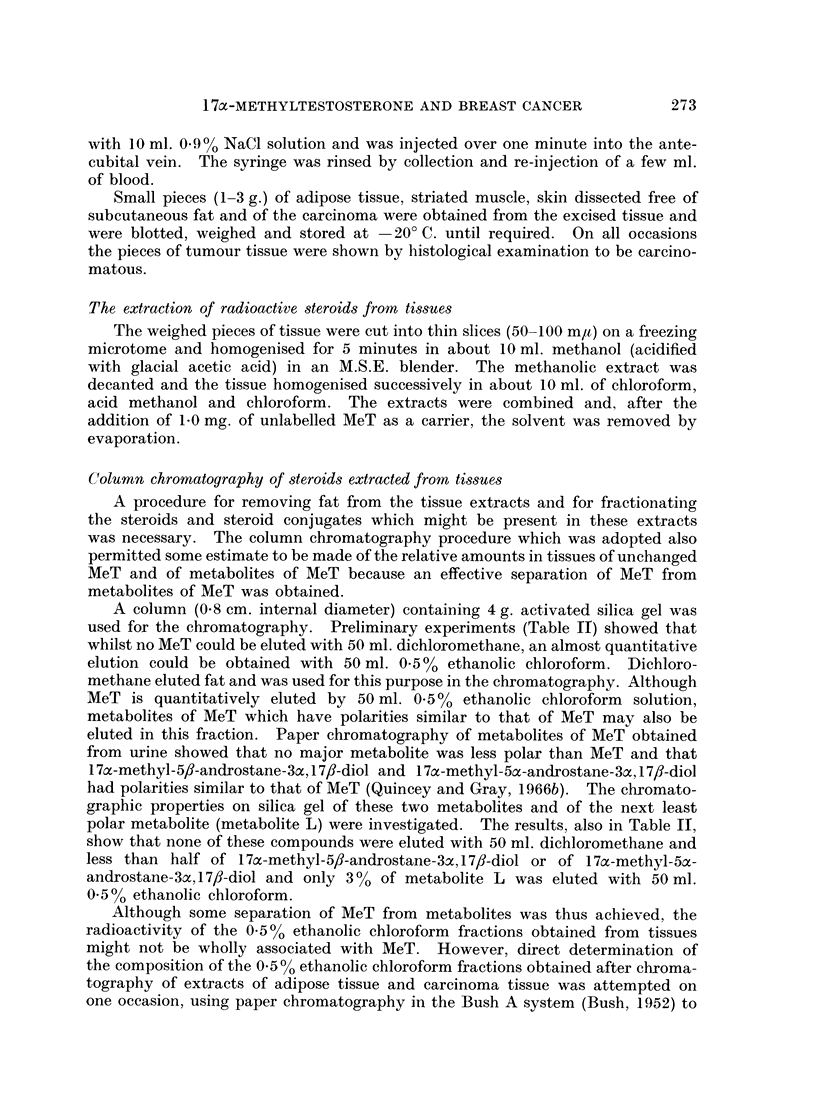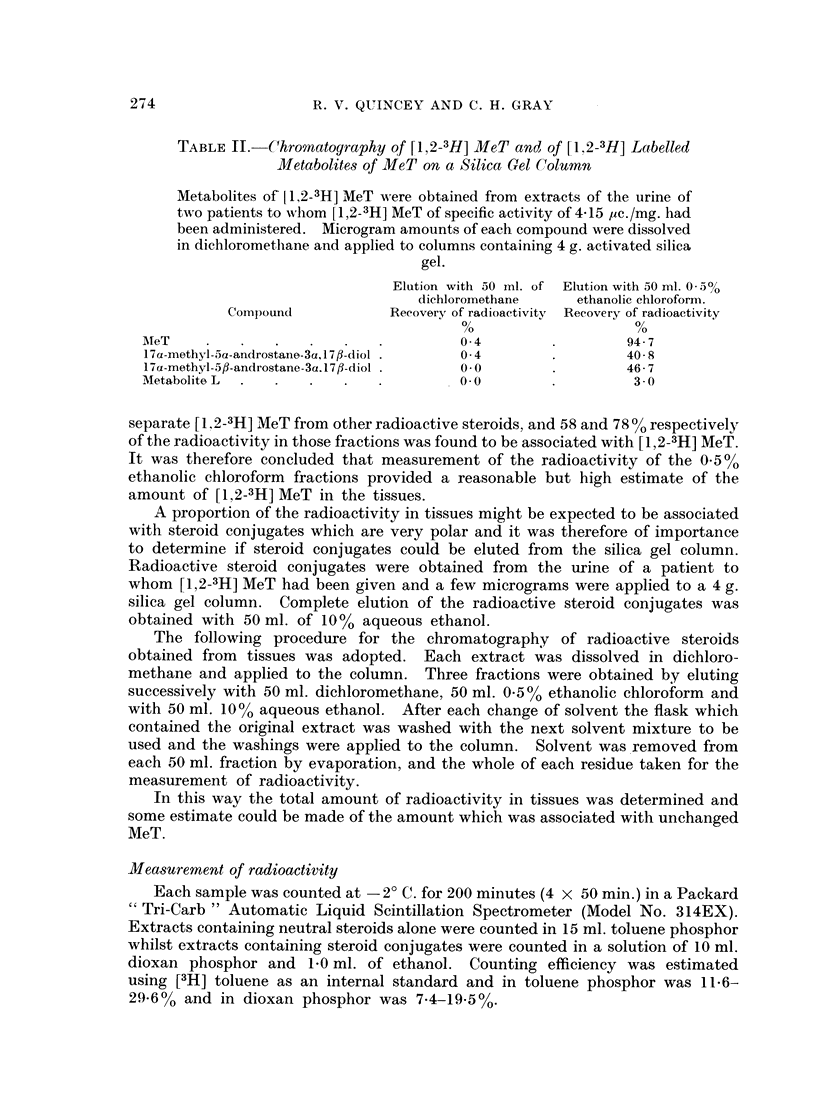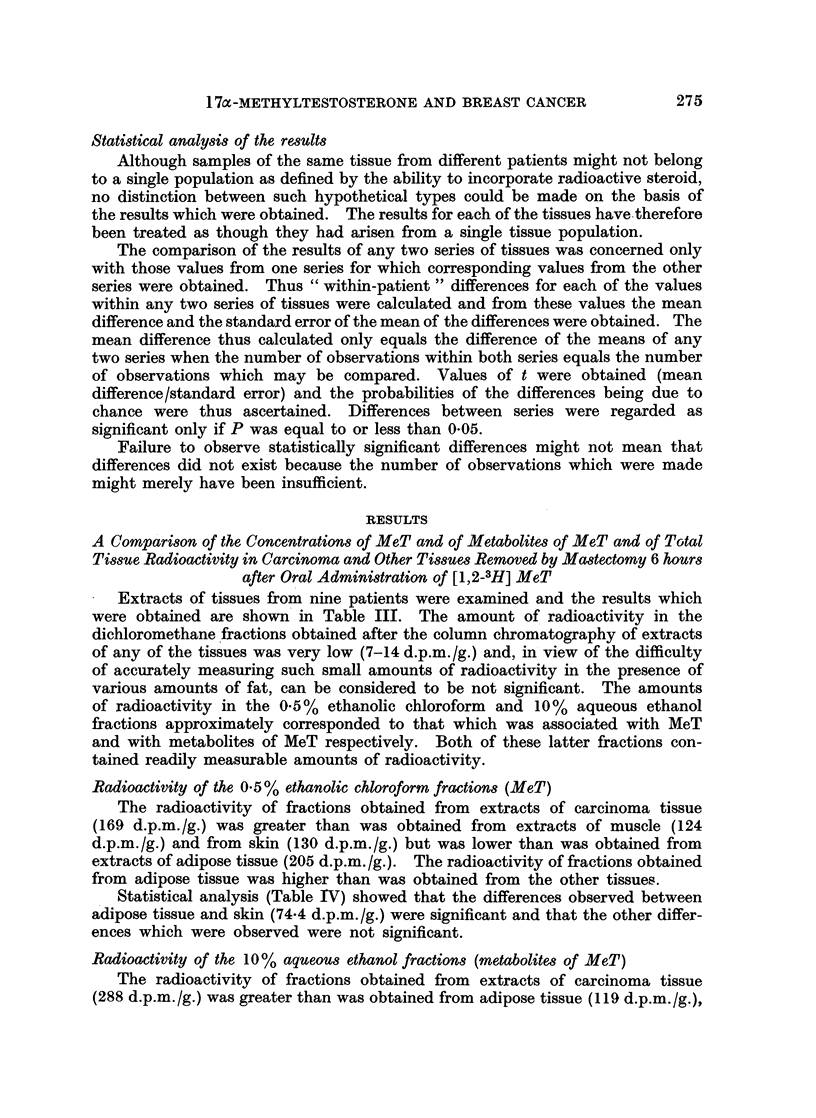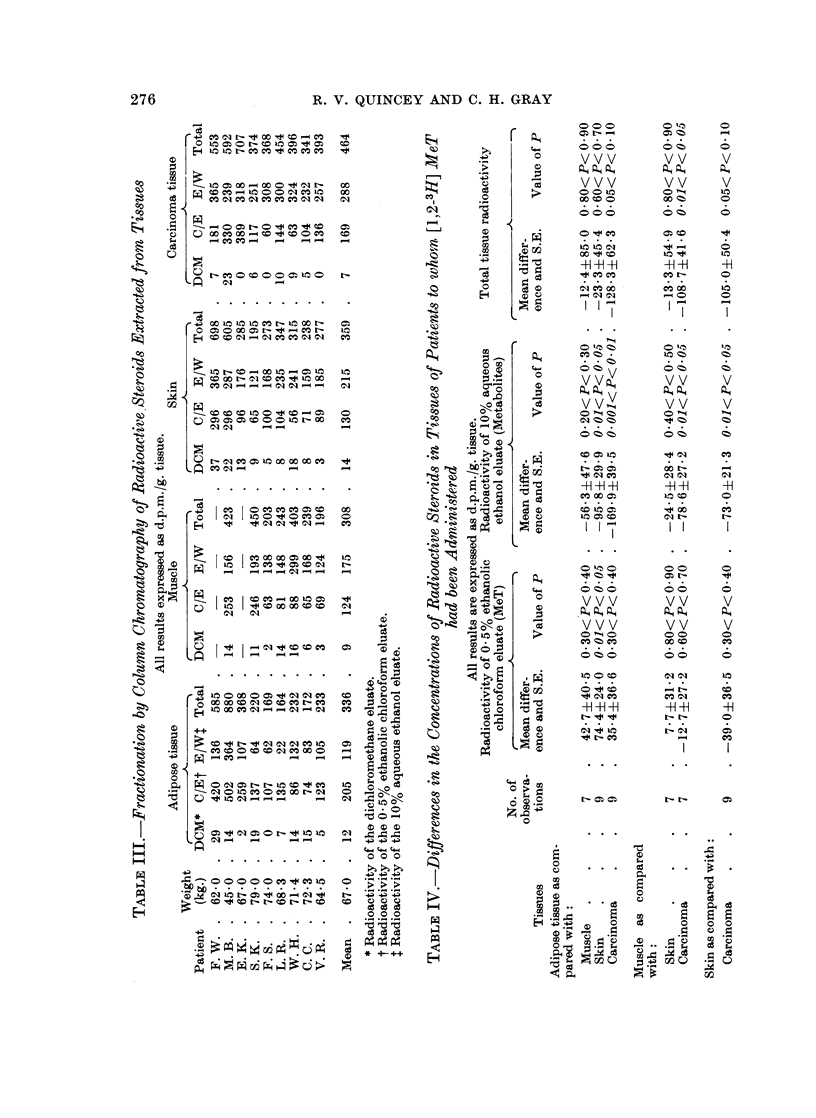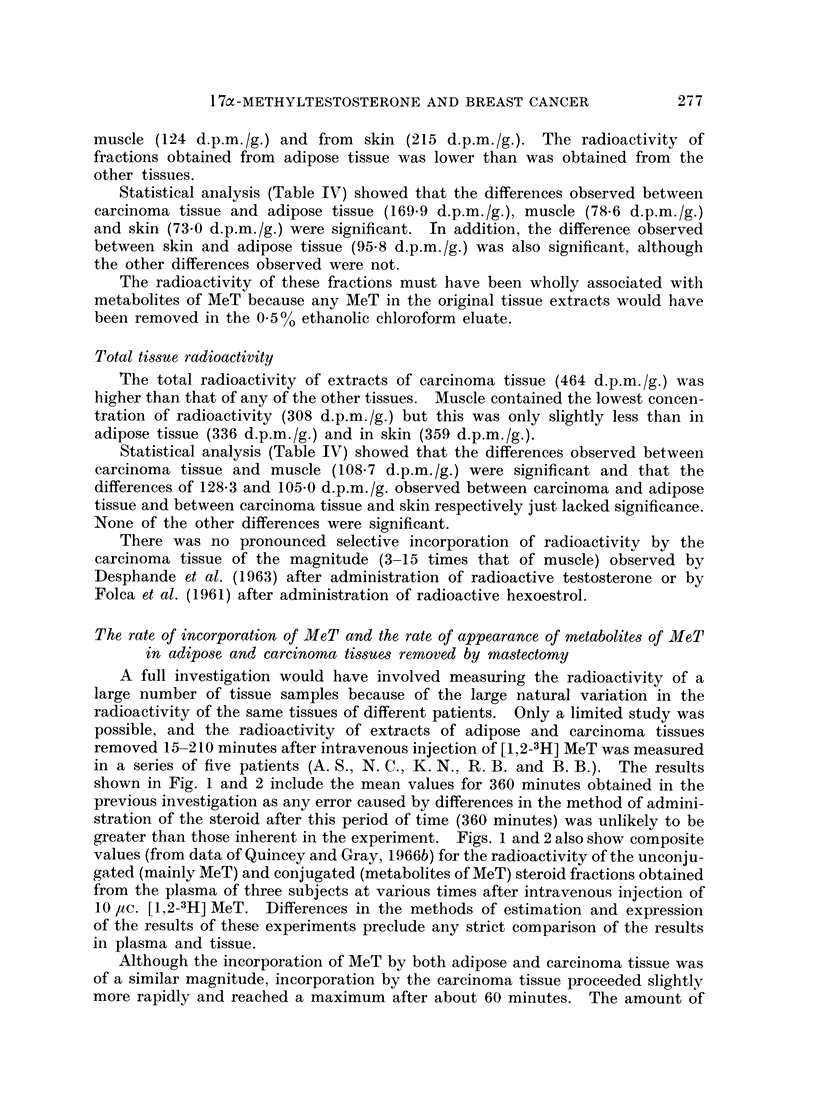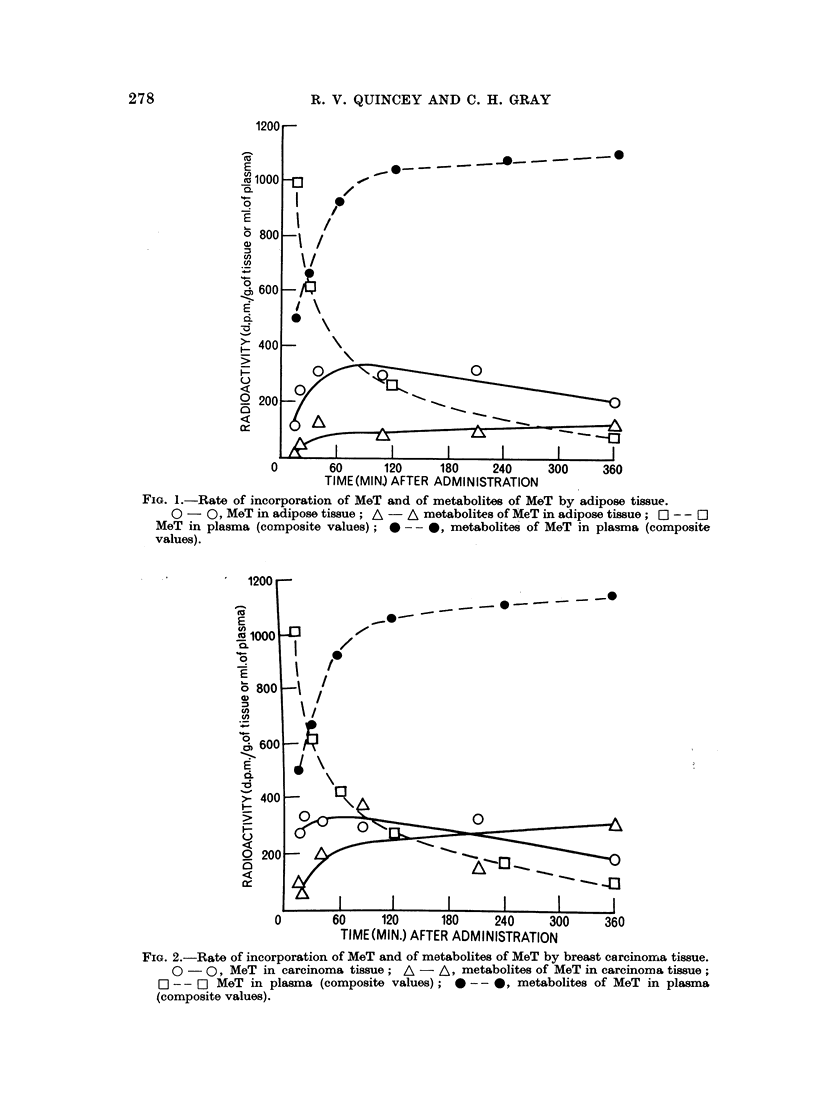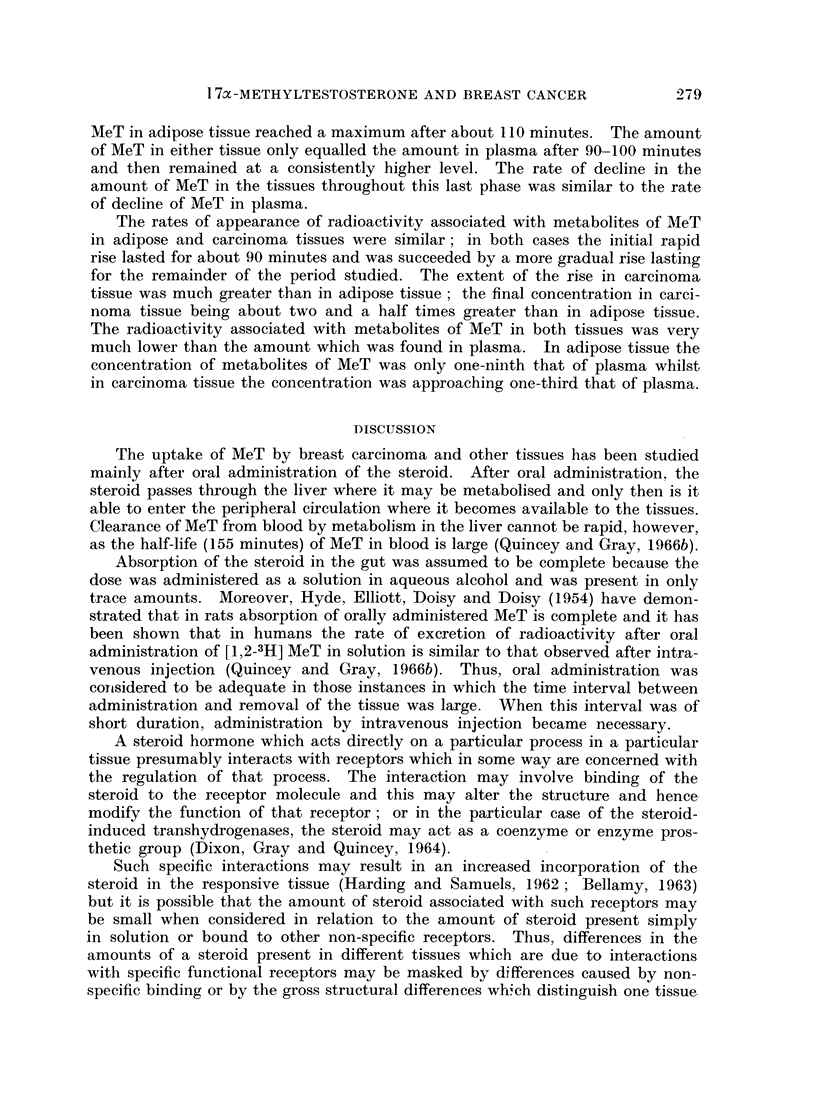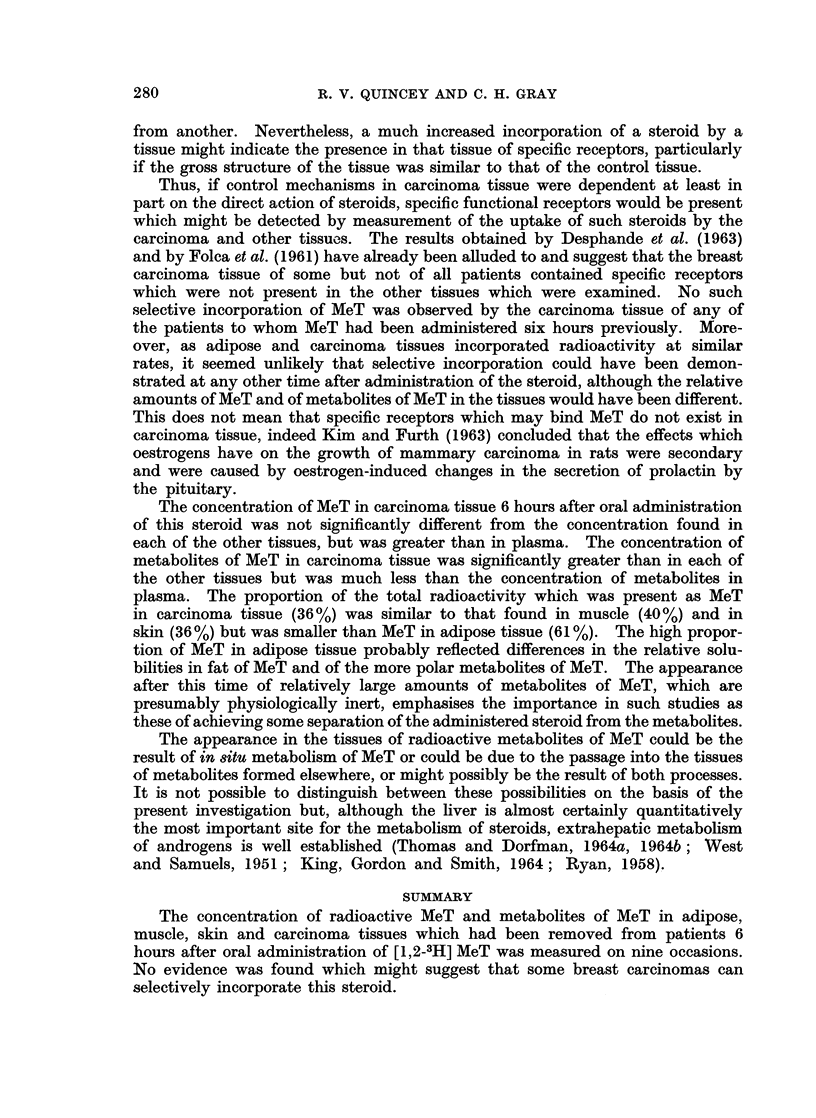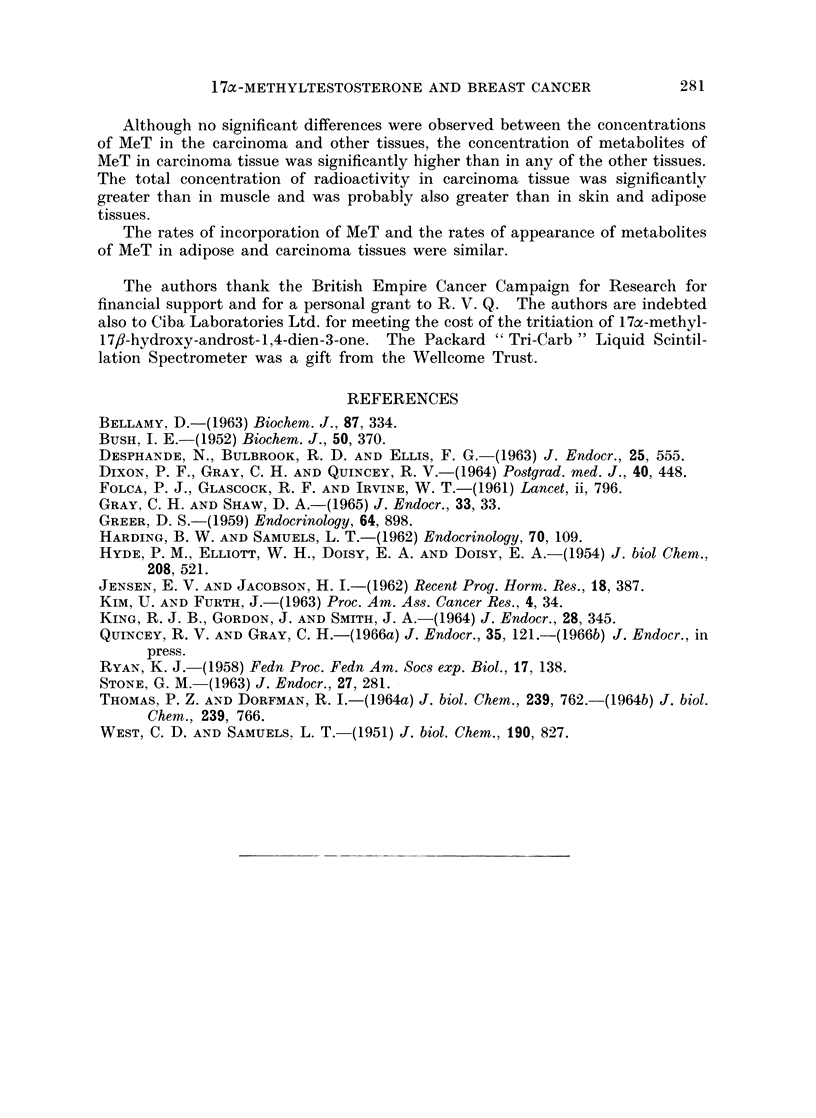# Uptake of [1,2-3H]17-alpha-methyltestosterone by breast carcinoma and other tissues of human subjects.

**DOI:** 10.1038/bjc.1966.35

**Published:** 1966-06

**Authors:** R. V. Quincey, C. H. Gray


### UPTAKE OF [1,2-$^3$H] 17$\alpha$-METHYLTESTOSTERONE BY BREAST CARCINOMA AND OTHER TISSUES OF HUMAN SUBJECTS

R. V. QUINCEY AND C. H. GRAY

*From the Department of Chemical Pathology, King's College Hospital Medical School, London, S.E.5*

Received for publication March 4, 1966

STUDIES *in vivo* on the incorporation of oestrogens by the uteri of immature rats (Jenson and Jacobson, 1962) and of ovariectomised mice (Stone, 1963), and on the incorporation of radioactivity by the ventral prostate and seminal vesicles of the male rat after injection of [$^{14}$C] testosterone (Greer, 1959) suggest that steroids may be selectively incorporated by those tissues whose growth is dependent on their presence. Such steroids might also be selectively incorporated by those breast carcinomas of humans which are influenced by steroids.

Evidence of a link in humans between the " hormone-dependency " of breast carcinomas and the ability of such carcinomas to incorporate hexoestrol (3,4-bis-(parahydroxy-phenyl)-n-hexane) was obtained by Folca, Glascock and Irvine (1961). In those patients who subsequently responded favourably to bilateral adrenalectomy and oophorectomy there was a selective incorporation of radioactivity by breast metastases removed 6 hours after intravenous injection of tritiated hexoestrol. More recently, Desphande, Bulbrook and Ellis (1963) found that 6 hours after intravenous injection of tritiated testosterone there had been selective incorporation of radioactivity by the breast carcinoma tissue of some, but not of other patients. The results of both these experiments suggest the existence of more than one class of breast carcinoma which differ in their ability to incorporate steroids. Since 17$\alpha$-methyltestosterone (MeT) is used for treatment of carcinoma of the breast it was thought that a similar investigation but concerned with the uptake of MeT by breast carcinoma and other tissues would be of importance. Determination of the concentration of radioactivity alone in carcinoma and in other tissues, however, does not necessarily permit a true comparison to be made of the relative amounts of the administered steroid present in the tissues because radioactive metabolites will almost certainly also be present. An attempt was made, therefore, to determine not only the total radioactivity of the tissues but also to determine the amount which was associated with unchanged MeT.

The amount of radioactivity in each tissue was expressed in terms of the wet weight of the tissue, although it is recognised that such a method takes no account of the many differences in the structures of those tissues which were examined.

A limited study of the rate of incorporation of MeT (and of radioactivity derived therefrom) by adipose and carcinoma tissues was also undertaken.

### MATERIALS

### [1,2-$^3$H] *MeT*

[1,2-$^3$H] MeT was prepared by the partial reduction of 17$\alpha$-methyl-17$\beta$-hydroxy-androst-1,4-dien-3-one in a tritium/hydrogen mixture with palladium on charcoal as the catalyst and, after purification, the steroid was diluted with unlabelled material to a specific activity of 1·0 $\mu$c./$\mu$g. (Quincey and Gray, 1966*a*). 10 $\mu$c. portions of the steroid were sealed in glass ampoules in 0·2 ml. ethanol and stored at $-20°$ C. Ampoules which had been stored for more than four months were not used.

*The subjects of investigation*

Each of the fourteen patients who were studied had an adenocarcinoma of the breast and all except one were women aged between 38 and 66 years. The exception was a male aged 67 years.

Clinical details relating to these patients are summarised in Table I.

TABLE I.—*Clinical Data of Patients in whom the Uptake of* [1,2-$^3H$ ] *MeT by Tissues Was Studied*

| Patient | Age years | Description of adenocarcinoma | Lymph node metastases |
|---|---|---|---|
| F. W. | 52 | Poorly-differentiated, poorly cellular | present |
| M. B. | 45 | Poorly-differentiated, cellular | present |
| E. K. | 54 | Poorly-differentiated, poorly cellular | present |
| S. K. | 56 | Poorly-differentiated, poorly cellular | present |
| F. S. | 61 | Poorly-differentiated, moderately cellular | absent |
| L. R. | 57 | Poorly-differentiated, poorly cellular | absent |
| W. H. | 53 | Poorly-differentiated, cellular | absent |
| C. C.* | 67 | Well-differentiated, cellular | absent |
| V. R. | 47 | Moderately well-differentiated, poorly cellular | present |
| A. S. | 38 | Poorly-differentiated, moderately cellular | absent |
| N. C. | 57 | Poorly-differentiated, cellular | present |
| K. N. | 66 | Poorly-differentiated, cellular | present |
| R. B. | 55 | Moderately well-differentiated, cellular | absent |
| B. B. | 66 | Well-differentiated, cellular | absent |

* Male

*Silica gel*

" M.F.C. " grade (Hopkins & Williams, Ltd.) was refluxed for 1 hour with concentrated aqueous ammonia to break down metal complexes, washed thoroughly with water and refluxed for 1 hour with 36N hydrochloric acid. It was then washed with water, methanol and chloroform and activated by heating for 16 hours at 120° C.

*Liquid scintillation phosphors*

Two phosphors based respectively on toluene and on dioxan were used; their composition is described by Gray and Shaw (1965).

*Solvents*

All solvents were of reagent grade and were redistilled before use.

### METHODS

*Administration of* [1,2-$^3H$] *MeT and the collection and storage of samples*

Each patient received 10 $\mu$c. [1,2-$^3$H] MeT. Steroid to be taken orally was mixed with 5 ml. ethanol, 15 ml. water and 5 ml. of orange juice immediately before administration. Steroid to be given by intravenous injection was mixed with 10 ml. 0·9% NaCl solution and was injected over one minute into the antecubital vein. The syringe was rinsed by collection and re-injection of a few ml. of blood.

Small pieces (1–3 g.) of adipose tissue, striated muscle, skin dissected free of subcutaneous fat and of the carcinoma were obtained from the excised tissue and were blotted, weighed and stored at −20° C. until required. On all occasions the pieces of tumour tissue were shown by histological examination to be carcinomatous.

*The extraction of radioactive steroids from tissues*

The weighed pieces of tissue were cut into thin slices (50–100 mμ) on a freezing microtome and homogenised for 5 minutes in about 10 ml. methanol (acidified with glacial acetic acid) in an M.S.E. blender. The methanolic extract was decanted and the tissue homogenised successively in about 10 ml. of chloroform, acid methanol and chloroform. The extracts were combined and, after the addition of 1·0 mg. of unlabelled MeT as a carrier, the solvent was removed by evaporation.

*Column chromatography of steroids extracted from tissues*

A procedure for removing fat from the tissue extracts and for fractionating the steroids and steroid conjugates which might be present in these extracts was necessary. The column chromatography procedure which was adopted also permitted some estimate to be made of the relative amounts in tissues of unchanged MeT and of metabolites of MeT because an effective separation of MeT from metabolites of MeT was obtained.

A column (0·8 cm. internal diameter) containing 4 g. activated silica gel was used for the chromatography. Preliminary experiments (Table II) showed that whilst no MeT could be eluted with 50 ml. dichloromethane, an almost quantitative elution could be obtained with 50 ml. 0·5% ethanolic chloroform. Dichloromethane eluted fat and was used for this purpose in the chromatography. Although MeT is quantitatively eluted by 50 ml. 0·5% ethanolic chloroform solution, metabolites of MeT which have polarities similar to that of MeT may also be eluted in this fraction. Paper chromatography of metabolites of MeT obtained from urine showed that no major metabolite was less polar than MeT and that 17α-methyl-5β-androstane-3α,17β-diol and 17α-methyl-5α-androstane-3α,17β-diol had polarities similar to that of MeT (Quincey and Gray, 1966*b*). The chromatographic properties on silica gel of these two metabolites and of the next least polar metabolite (metabolite L) were investigated. The results, also in Table II, show that none of these compounds were eluted with 50 ml. dichloromethane and less than half of 17α-methyl-5β-androstane-3α,17β-diol or of 17α-methyl-5α-androstane-3α,17β-diol and only 3% of metabolite L was eluted with 50 ml. 0·5% ethanolic chloroform.

Although some separation of MeT from metabolites was thus achieved, the radioactivity of the 0·5% ethanolic chloroform fractions obtained from tissues might not be wholly associated with MeT. However, direct determination of the composition of the 0·5% ethanolic chloroform fractions obtained after chromatography of extracts of adipose tissue and carcinoma tissue was attempted on one occasion, using paper chromatography in the Bush A system (Bush, 1952) to

TABLE II.—*Chromatography of [1,2-$^3$H] MeT and of [1,2-$^3$H] Labelled Metabolites of MeT on a Silica Gel Column*

Metabolites of [1,2-$^3$H] MeT were obtained from extracts of the urine of two patients to whom [1,2-$^3$H] MeT of specific activity of 4·15 $\mu$c./mg. had been administered. Microgram amounts of each compound were dissolved in dichloromethane and applied to columns containing 4 g. activated silica gel.

| Compound | Elution with 50 ml. of dichloromethane Recovery of radioactivity % | Elution with 50 ml. 0·5% ethanolic chloroform. Recovery of radioactivity % |
|---|---|---|
| MeT | 0·4 | 94·7 |
| 17$\alpha$-methyl-5$\alpha$-androstane-3$\alpha$,17$\beta$-diol | 0·4 | 40·8 |
| 17$\alpha$-methyl-5$\beta$-androstane-3$\alpha$,17$\beta$-diol | 0·0 | 46·7 |
| Metabolite L | 0·0 | 3·0 |

separate [1,2-$^3$H] MeT from other radioactive steroids, and 58 and 78% respectively of the radioactivity in those fractions was found to be associated with [1,2-$^3$H] MeT. It was therefore concluded that measurement of the radioactivity of the 0·5% ethanolic chloroform fractions provided a reasonable but high estimate of the amount of [1,2-$^3$H] MeT in the tissues.

A proportion of the radioactivity in tissues might be expected to be associated with steroid conjugates which are very polar and it was therefore of importance to determine if steroid conjugates could be eluted from the silica gel column. Radioactive steroid conjugates were obtained from the urine of a patient to whom [1,2-$^3$H] MeT had been given and a few micrograms were applied to a 4 g. silica gel column. Complete elution of the radioactive steroid conjugates was obtained with 50 ml. of 10% aqueous ethanol.

The following procedure for the chromatography of radioactive steroids obtained from tissues was adopted. Each extract was dissolved in dichloromethane and applied to the column. Three fractions were obtained by eluting successively with 50 ml. dichloromethane, 50 ml. 0·5% ethanolic chloroform and with 50 ml. 10% aqueous ethanol. After each change of solvent the flask which contained the original extract was washed with the next solvent mixture to be used and the washings were applied to the column. Solvent was removed from each 50 ml. fraction by evaporation, and the whole of each residue taken for the measurement of radioactivity.

In this way the total amount of radioactivity in tissues was determined and some estimate could be made of the amount which was associated with unchanged MeT.

*Measurement of radioactivity*

Each sample was counted at $-2°$ C. for 200 minutes (4 $\times$ 50 min.) in a Packard "Tri-Carb" Automatic Liquid Scintillation Spectrometer (Model No. 314EX). Extracts containing neutral steroids alone were counted in 15 ml. toluene phosphor whilst extracts containing steroid conjugates were counted in a solution of 10 ml. dioxan phosphor and 1·0 ml. of ethanol. Counting efficiency was estimated using [$^3$H] toluene as an internal standard and in toluene phosphor was 11·6–29·6% and in dioxan phosphor was 7·4–19·5%.

*Statistical analysis of the results*

Although samples of the same tissue from different patients might not belong to a single population as defined by the ability to incorporate radioactive steroid, no distinction between such hypothetical types could be made on the basis of the results which were obtained. The results for each of the tissues have therefore been treated as though they had arisen from a single tissue population.

The comparison of the results of any two series of tissues was concerned only with those values from one series for which corresponding values from the other series were obtained. Thus " within-patient " differences for each of the values within any two series of tissues were calculated and from these values the mean difference and the standard error of the mean of the differences were obtained. The mean difference thus calculated only equals the difference of the means of any two series when the number of observations within both series equals the number of observations which may be compared. Values of $t$ were obtained (mean difference/standard error) and the probabilities of the differences being due to chance were thus ascertained. Differences between series were regarded as significant only if $P$ was equal to or less than 0·05.

Failure to observe statistically significant differences might not mean that differences did not exist because the number of observations which were made might merely have been insufficient.

### RESULTS

*A Comparison of the Concentrations of MeT and of Metabolites of MeT and of Total Tissue Radioactivity in Carcinoma and Other Tissues Removed by Mastectomy* 6 *hours after Oral Administration of* [1,2-$^3$*H*] *MeT*

Extracts of tissues from nine patients were examined and the results which were obtained are shown in Table III. The amount of radioactivity in the dichloromethane fractions obtained after the column chromatography of extracts of any of the tissues was very low (7–14 d.p.m./g.) and, in view of the difficulty of accurately measuring such small amounts of radioactivity in the presence of various amounts of fat, can be considered to be not significant. The amounts of radioactivity in the 0·5% ethanolic chloroform and 10% aqueous ethanol fractions approximately corresponded to that which was associated with MeT and with metabolites of MeT respectively. Both of these latter fractions contained readily measurable amounts of radioactivity.

*Radioactivity of the* 0·5% *ethanolic chloroform fractions* (*MeT*)

The radioactivity of fractions obtained from extracts of carcinoma tissue (169 d.p.m./g.) was greater than was obtained from extracts of muscle (124 d.p.m./g.) and from skin (130 d.p.m./g.) but was lower than was obtained from extracts of adipose tissue (205 d.p.m./g.). The radioactivity of fractions obtained from adipose tissue was higher than was obtained from the other tissues.

Statistical analysis (Table IV) showed that the differences observed between adipose tissue and skin (74·4 d.p.m./g.) were significant and that the other differences which were observed were not significant.

*Radioactivity of the* 10% *aqueous ethanol fractions* (*metabolites of MeT*)

The radioactivity of fractions obtained from extracts of carcinoma tissue (288 d.p.m./g.) was greater than was obtained from adipose tissue (119 d.p.m./g.),

TABLE III.—*Fractionation by Column Chromatography of Radioactive Steroids Extracted from Tissues*

All results expressed as d.p.m./g. tissue.

| Patient | Weight (kg.) | Adipose tissue DCM* | Adipose tissue C/E† | Adipose tissue E/W‡ | Adipose tissue Total | Muscle DCM | Muscle C/E | Muscle E/W | Muscle Total | Skin DCM | Skin C/E | Skin E/W | Skin Total | Carcinoma tissue DCM | Carcinoma tissue C/E | Carcinoma tissue E/W | Carcinoma tissue Total |
|---|---|---|---|---|---|---|---|---|---|---|---|---|---|---|---|---|---|
| F. W. | 62·0 | 29 | 420 | 136 | 585 | — | — | — | — | 37 | 296 | 365 | 698 | 7 | 181 | 365 | 553 |
| M. B. | 45·0 | 14 | 502 | 364 | 880 | 14 | 253 | 156 | 423 | 22 | 296 | 287 | 605 | 23 | 330 | 239 | 592 |
| E. K. | 67·0 | 2 | 259 | 107 | 368 | — | — | — | — | 13 | 96 | 176 | 285 | 0 | 389 | 318 | 707 |
| S. K. | 79·0 | 19 | 137 | 64 | 220 | 11 | 246 | 193 | 450 | 9 | 65 | 121 | 195 | 6 | 117 | 251 | 374 |
| F. S. | 74·0 | 0 | 107 | 62 | 169 | 2 | 63 | 138 | 203 | 5 | 100 | 168 | 273 | 0 | 60 | 308 | 368 |
| L. R. | 68·3 | 7 | 135 | 22 | 164 | 14 | 81 | 148 | 243 | 8 | 104 | 235 | 347 | 10 | 144 | 300 | 454 |
| W. H. | 71·4 | 14 | 86 | 132 | 232 | 16 | 88 | 299 | 403 | 18 | 56 | 241 | 315 | 9 | 63 | 324 | 396 |
| C. C. | 72·3 | 15 | 74 | 83 | 172 | 6 | 65 | 168 | 239 | 8 | 71 | 159 | 238 | 5 | 104 | 232 | 341 |
| V. R. | 64·5 | 5 | 123 | 105 | 233 | 3 | 69 | 124 | 196 | 3 | 89 | 185 | 277 | 0 | 136 | 257 | 393 |
| Mean | 67·0 | 12 | 205 | 119 | 336 | 9 | 124 | 175 | 308 | 14 | 130 | 215 | 359 | 7 | 169 | 288 | 464 |

* Radioactivity of the dichloromethane eluate.
† Radioactivity of the 0·5% ethanolic chloroform eluate.
‡ Radioactivity of the 10% aqueous ethanol eluate.

TABLE IV.—*Differences in the Concentrations of Radioactive Steroids in Tissues of Patients to whom* [1,2-$^3H$] *MeT had been Administered*

All results are expressed as d.p.m./g. tissue.

| Tissues | No. of observations | Radioactivity of 0·5% ethanolic chloroform eluate (MeT): Mean difference and S.E. | Radioactivity of 0·5% ethanolic chloroform eluate (MeT): Value of $P$ | Radioactivity of 10% aqueous ethanol eluate (Metabolites): Mean difference and S.E. | Radioactivity of 10% aqueous ethanol eluate (Metabolites): Value of $P$ | Total tissue radioactivity: Mean difference and S.E. | Total tissue radioactivity: Value of $P$ |
|---|---|---|---|---|---|---|---|
| Adipose tissue as compared with : | | | | | | | |
| Muscle | 7 | 42·7±40·5 | 0·30<$P$<0·40 | −56·3±47·6 | 0·20<$P$<0·30 | −12·4±85·0 | 0·80<$P$<0·90 |
| Skin | 9 | 74·4±24·0 | *0·01<$P$<0·05* | −95·8±29·9 | *0·01<$P$<0·05* | −23·3±45·4 | 0·60<$P$<0·70 |
| Carcinoma | 9 | 35·4±36·6 | 0·30<$P$<0·40 | −169·9±39·5 | *0·001<$P$<0·01* | −128·3±62·3 | 0·05<$P$<0·10 |
| Muscle as compared with : | | | | | | | |
| Skin | 7 | 7·7±31·2 | 0·80<$P$<0·90 | −24·5±28·4 | 0·40<$P$<0·50 | −13·3±54·9 | 0·80<$P$<0·90 |
| Carcinoma | 7 | −12·7±27·2 | 0·60<$P$<0·70 | −78·6±27·2 | *0·01<$P$<0·05* | −108·7±41·6 | *0·01<$P$<0·05* |
| Skin as compared with : | | | | | | | |
| Carcinoma | 9 | −39·0±36·5 | 0·30<$P$<0·40 | −73·0±21·3 | *0·01<$P$<0·05* | −105·0±50·4 | 0·05<$P$<0·10 |

muscle (124 d.p.m./g.) and from skin (215 d.p.m./g.). The radioactivity of fractions obtained from adipose tissue was lower than was obtained from the other tissues.

Statistical analysis (Table IV) showed that the differences observed between carcinoma tissue and adipose tissue (169·9 d.p.m./g.), muscle (78·6 d.p.m./g.) and skin (73·0 d.p.m./g.) were significant. In addition, the difference observed between skin and adipose tissue (95·8 d.p.m./g.) was also significant, although the other differences observed were not.

The radioactivity of these fractions must have been wholly associated with metabolites of MeT because any MeT in the original tissue extracts would have been removed in the 0·5% ethanolic chloroform eluate.

*Total tissue radioactivity*

The total radioactivity of extracts of carcinoma tissue (464 d.p.m./g.) was higher than that of any of the other tissues. Muscle contained the lowest concentration of radioactivity (308 d.p.m./g.) but this was only slightly less than in adipose tissue (336 d.p.m./g.) and in skin (359 d.p.m./g.).

Statistical analysis (Table IV) showed that the differences observed between carcinoma tissue and muscle (108·7 d.p.m./g.) were significant and that the differences of 128·3 and 105·0 d.p.m./g. observed between carcinoma and adipose tissue and between carcinoma tissue and skin respectively just lacked significance. None of the other differences were significant.

There was no pronounced selective incorporation of radioactivity by the carcinoma tissue of the magnitude (3–15 times that of muscle) observed by Desphande *et al.* (1963) after administration of radioactive testosterone or by Folca *et al.* (1961) after administration of radioactive hexoestrol.

*The rate of incorporation of MeT and the rate of appearance of metabolites of MeT in adipose and carcinoma tissues removed by mastectomy*

A full investigation would have involved measuring the radioactivity of a large number of tissue samples because of the large natural variation in the radioactivity of the same tissues of different patients. Only a limited study was possible, and the radioactivity of extracts of adipose and carcinoma tissues removed 15–210 minutes after intravenous injection of [1,2-$^{3}$H] MeT was measured in a series of five patients (A. S., N. C., K. N., R. B. and B. B.). The results shown in Fig. 1 and 2 include the mean values for 360 minutes obtained in the previous investigation as any error caused by differences in the method of administration of the steroid after this period of time (360 minutes) was unlikely to be greater than those inherent in the experiment. Figs. 1 and 2 also show composite values (from data of Quincey and Gray, 1966*b*) for the radioactivity of the unconjugated (mainly MeT) and conjugated (metabolites of MeT) steroid fractions obtained from the plasma of three subjects at various times after intravenous injection of 10 $\mu$c. [1,2-$^{3}$H] MeT. Differences in the methods of estimation and expression of the results of these experiments preclude any strict comparison of the results in plasma and tissue.

Although the incorporation of MeT by both adipose and carcinoma tissue was of a similar magnitude, incorporation by the carcinoma tissue proceeded slightly more rapidly and reached a maximum after about 60 minutes. The amount of

FIG. 1.—Rate of incorporation of MeT and of metabolites of MeT by adipose tissue. ○ — ○, MeT in adipose tissue ; △ — △ metabolites of MeT in adipose tissue ; □ -- □ MeT in plasma (composite values) ; ● -- ●, metabolites of MeT in plasma (composite values).

FIG. 2.—Rate of incorporation of MeT and of metabolites of MeT by breast carcinoma tissue. ○ — ○, MeT in carcinoma tissue ; △ — △, metabolites of MeT in carcinoma tissue ; □ -- □ MeT in plasma (composite values) ; ● -- ●, metabolites of MeT in plasma (composite values).

MeT in adipose tissue reached a maximum after about 110 minutes. The amount of MeT in either tissue only equalled the amount in plasma after 90–100 minutes and then remained at a consistently higher level. The rate of decline in the amount of MeT in the tissues throughout this last phase was similar to the rate of decline of MeT in plasma.

The rates of appearance of radioactivity associated with metabolites of MeT in adipose and carcinoma tissues were similar; in both cases the initial rapid rise lasted for about 90 minutes and was succeeded by a more gradual rise lasting for the remainder of the period studied. The extent of the rise in carcinoma tissue was much greater than in adipose tissue; the final concentration in carcinoma tissue being about two and a half times greater than in adipose tissue. The radioactivity associated with metabolites of MeT in both tissues was very much lower than the amount which was found in plasma. In adipose tissue the concentration of metabolites of MeT was only one-ninth that of plasma whilst in carcinoma tissue the concentration was approaching one-third that of plasma.

### DISCUSSION

The uptake of MeT by breast carcinoma and other tissues has been studied mainly after oral administration of the steroid. After oral administration, the steroid passes through the liver where it may be metabolised and only then is it able to enter the peripheral circulation where it becomes available to the tissues. Clearance of MeT from blood by metabolism in the liver cannot be rapid, however, as the half-life (155 minutes) of MeT in blood is large (Quincey and Gray, 1966*b*).

Absorption of the steroid in the gut was assumed to be complete because the dose was administered as a solution in aqueous alcohol and was present in only trace amounts. Moreover, Hyde, Elliott, Doisy and Doisy (1954) have demonstrated that in rats absorption of orally administered MeT is complete and it has been shown that in humans the rate of excretion of radioactivity after oral administration of [1,2-$^{3}$H] MeT in solution is similar to that observed after intravenous injection (Quincey and Gray, 1966*b*). Thus, oral administration was considered to be adequate in those instances in which the time interval between administration and removal of the tissue was large. When this interval was of short duration, administration by intravenous injection became necessary.

A steroid hormone which acts directly on a particular process in a particular tissue presumably interacts with receptors which in some way are concerned with the regulation of that process. The interaction may involve binding of the steroid to the receptor molecule and this may alter the structure and hence modify the function of that receptor; or in the particular case of the steroid-induced transhydrogenases, the steroid may act as a coenzyme or enzyme prosthetic group (Dixon, Gray and Quincey, 1964).

Such specific interactions may result in an increased incorporation of the steroid in the responsive tissue (Harding and Samuels, 1962; Bellamy, 1963) but it is possible that the amount of steroid associated with such receptors may be small when considered in relation to the amount of steroid present simply in solution or bound to other non-specific receptors. Thus, differences in the amounts of a steroid present in different tissues which are due to interactions with specific functional receptors may be masked by differences caused by non-specific binding or by the gross structural differences which distinguish one tissue from another. Nevertheless, a much increased incorporation of a steroid by a tissue might indicate the presence in that tissue of specific receptors, particularly if the gross structure of the tissue was similar to that of the control tissue.

Thus, if control mechanisms in carcinoma tissue were dependent at least in part on the direct action of steroids, specific functional receptors would be present which might be detected by measurement of the uptake of such steroids by the carcinoma and other tissues. The results obtained by Desphande *et al.* (1963) and by Folca *et al.* (1961) have already been alluded to and suggest that the breast carcinoma tissue of some but not of all patients contained specific receptors which were not present in the other tissues which were examined. No such selective incorporation of MeT was observed by the carcinoma tissue of any of the patients to whom MeT had been administered six hours previously. Moreover, as adipose and carcinoma tissues incorporated radioactivity at similar rates, it seemed unlikely that selective incorporation could have been demonstrated at any other time after administration of the steroid, although the relative amounts of MeT and of metabolites of MeT in the tissues would have been different. This does not mean that specific receptors which may bind MeT do not exist in carcinoma tissue, indeed Kim and Furth (1963) concluded that the effects which oestrogens have on the growth of mammary carcinoma in rats were secondary and were caused by oestrogen-induced changes in the secretion of prolactin by the pituitary.

The concentration of MeT in carcinoma tissue 6 hours after oral administration of this steroid was not significantly different from the concentration found in each of the other tissues, but was greater than in plasma. The concentration of metabolites of MeT in carcinoma tissue was significantly greater than in each of the other tissues but was much less than the concentration of metabolites in plasma. The proportion of the total radioactivity which was present as MeT in carcinoma tissue (36%) was similar to that found in muscle (40%) and in skin (36%) but was smaller than MeT in adipose tissue (61%). The high proportion of MeT in adipose tissue probably reflected differences in the relative solubilities in fat of MeT and of the more polar metabolites of MeT. The appearance after this time of relatively large amounts of metabolites of MeT, which are presumably physiologically inert, emphasises the importance in such studies as these of achieving some separation of the administered steroid from the metabolites.

The appearance in the tissues of radioactive metabolites of MeT could be the result of *in situ* metabolism of MeT or could be due to the passage into the tissues of metabolites formed elsewhere, or might possibly be the result of both processes. It is not possible to distinguish between these possibilities on the basis of the present investigation but, although the liver is almost certainly quantitatively the most important site for the metabolism of steroids, extrahepatic metabolism of androgens is well established (Thomas and Dorfman, 1964*a*, 1964*b* ; West and Samuels, 1951 ; King, Gordon and Smith, 1964 ; Ryan, 1958).

### SUMMARY

The concentration of radioactive MeT and metabolites of MeT in adipose, muscle, skin and carcinoma tissues which had been removed from patients 6 hours after oral administration of [1,2-$^3$H] MeT was measured on nine occasions. No evidence was found which might suggest that some breast carcinomas can selectively incorporate this steroid.

Although no significant differences were observed between the concentrations of MeT in the carcinoma and other tissues, the concentration of metabolites of MeT in carcinoma tissue was significantly higher than in any of the other tissues. The total concentration of radioactivity in carcinoma tissue was significantly greater than in muscle and was probably also greater than in skin and adipose tissues.

The rates of incorporation of MeT and the rates of appearance of metabolites of MeT in adipose and carcinoma tissues were similar.

The authors thank the British Empire Cancer Campaign for Research for financial support and for a personal grant to R. V. Q. The authors are indebted also to Ciba Laboratories Ltd. for meeting the cost of the tritiation of 17α-methyl-17β-hydroxy-androst-1,4-dien-3-one. The Packard " Tri-Carb " Liquid Scintillation Spectrometer was a gift from the Wellcome Trust.

### REFERENCES

BELLAMY, D.—(1963) *Biochem. J.*, **87**, 334.
BUSH, I. E.—(1952) *Biochem. J.*, **50**, 370.
DESPHANDE, N., BULBROOK, R. D. AND ELLIS, F. G.—(1963) *J. Endocr.*, **25**, 555.
DIXON, P. F., GRAY, C. H. AND QUINCEY, R. V.—(1964) *Postgrad. med. J.*, **40**, 448.
FOLCA, P. J., GLASCOCK, R. F. AND IRVINE, W. T.—(1961) *Lancet*, ii, 796.
GRAY, C. H. AND SHAW, D. A.—(1965) *J. Endocr.*, **33**, 33.
GREER, D. S.—(1959) *Endocrinology*, **64**, 898.
HARDING, B. W. AND SAMUELS, L. T.—(1962) *Endocrinology*, **70**, 109.
HYDE, P. M., ELLIOTT, W. H., DOISY, E. A. AND DOISY, E. A.—(1954) *J. biol Chem.*, **208**, 521.
JENSEN, E. V. AND JACOBSON, H. I.—(1962) *Recent Prog. Horm. Res.*, **18**, 387.
KIM, U. AND FURTH, J.—(1963) *Proc. Am. Ass. Cancer Res.*, **4**, 34.
KING, R. J. B., GORDON, J. AND SMITH, J. A.—(1964) *J. Endocr.*, **28**, 345.
QUINCEY, R. V. AND GRAY, C. H.—(1966*a*) *J. Endocr.*, **35**, 121.—(1966*b*) *J. Endocr.*, in press.
RYAN, K. J.—(1958) *Fedn Proc. Fedn Am. Socs exp. Biol.*, **17**, 138.
STONE, G. M.—(1963) *J. Endocr.*, **27**, 281.
THOMAS, P. Z. AND DORFMAN, R. I.—(1964*a*) *J. biol. Chem.*, **239**, 762.—(1964*b*) *J. biol. Chem.*, **239**, 766.
WEST, C. D. AND SAMUELS, L. T.—(1951) *J. biol. Chem.*, **190**, 827.